# Women Are Better at Selecting Gifts than Men

**DOI:** 10.1371/journal.pone.0081643

**Published:** 2013-12-26

**Authors:** Monique M. H. Pollmann, Ilja van Beest

**Affiliations:** 1 Department of Communication and Information Sciences, Tilburg University, Tilburg, The Netherlands; 2 Department of Social Psychology, Tilburg University, Tilburg, The Netherlands; California Pacific Medicial Center Research Institute, United States of America

## Abstract

There is a widespread belief that women are better at selecting gifts than men; however, this claim has not been assessed on the basis of objective criteria. The current studies do exactly that and show that women do indeed make better gift selections for others, regardless of the gender of the receiver and the type of relationship between the giver and receiver. We investigate the mediating role of different aspects of interpersonal sensitivity and reveal that differences in interpersonal interest (measured with an autism questionnaire), but not differences in interpersonal reactivity, explain gender differences in gift selection quality. The current studies thus present the first objective evidence for the claim that women are better in selecting gifts for others and also give an indication of why this is the case.

## Introduction

Gift giving is a universal phenomenon in almost every human culture. Every year, billions of dollars are spent on birthday and holiday gifts. It is estimated that roughly 10% of all sales at GAFO-type stores concern gifts for others [1]. Although meant as a sign of love or appreciation, not all gifts are valued as much as the giver wishes for [2]. Given the difficulty in selecting the perfect gift, it is important to investigate predictors of successful gift giving. One of the factors that is likely to play an important role in gift giving is the giver's gender. Women are thought to be the primary gift givers because they are more concerned about expressing love than men [3]. From this follows the widespread belief that women are *better* gift-givers than men [4]. But is this really the case, and if so, why? The current study investigates whether women are better at selecting gifts than men and investigates possible explanations for this effect.

### The importance of good gift selection

Given its universal nature, it is not surprising that gift giving has been studied from several different angles [5], [6], [7], [8]. It serves important interpersonal functions because gifts can help build and reformulate personal relationships [9], [10] and promote the giver's happiness [11]. However, gifts do not always have positive consequences. A recent experimental study showed that bad gifts can actually harm one's relationship [12]. In this study, romantic partners were led to believe that the other partner had selected a gift for them that either fit their preferences quite well or rather badly. Men who received bad gifts subsequently perceived less similarity with their partner and predicted their relationship would end sooner than men who received good gifts.

Thus, prior research has established that gift giving benefits the giver, benefits the receiver, and benefits the relationship, but that these effects depend on receiving the appropriate gift. It is therefore surprising that the conditions under which people select the right or the wrong gift are understudied.

In this set of studies, we focus on the objective quality of gift selection by comparing the preferences of a recipient with the actual selection of the giver. Note that we acknowledge that the evaluation of gifts can be influenced by other factors such as the relationship with the giver or the recipient's emotional state. However, everything being equal, we believe that a better match between preferences and selection is an objective indication of higher gift quality. Moreover, another benefit of this measure is that cannot be influenced by the hindsight of the recipient.

### Gender difference in gift selection

Are men or women better in selecting the right gift? Although no study so far has objectively tested whether women are better gift givers than men, there are some studies showing that women are more *involved* in gift giving than men. For example, [13] interviewed men and women about their Christmas shopping behavior and found that women report buying gifts for a greater number of recipients, starting shopping earlier, and spending more time per recipient when buying gifts. What is more, women estimate that 10 percent of the gifts they give are returned by the recipient, whereas men estimate that 16 percent of the gifts they give are returned. Based on these numbers, one can infer that women think that they are more successful gift-givers. However, self-reported skills do not always predict actual skills. For example, people's self-indicated ability to know and understand others' intentions and personality is often unrelated to actual levels of knowledge and understanding [14], [15]. Thus, whether women's self-reported superiority is based on selecting gifts that are actually has yet to be tested.

### Interpersonal interest or interpersonal reactivity?

If women are better at selecting gifts than men, what are possible explanations for this difference? We think that gender differences in interpersonal sensitivity are relevant in the gift-giving context. There are two factors that contribute to interpersonal sensitivity: (1) one needs to have interest in other people (interpersonal interest) and (2) one needs to be able to take the perspective of others (interpersonal reactivity). To the extent that a person is interested in other people and able to effectively take another person's perspective, they should better be able to predict a target's gift preferences.

We measure the first aspect, *interpersonal interest*, with the autism spectrum quotient (AQ) [16]. A key aspect of this questionnaire is that it captures the degree to which people care about social interactions relative to non-social interactions (e.g., ‘I enjoy social events’, ‘I feel more attracted to people than to objects’ (reverse coded)). In general, reduced interest in socially relevant information is an important aspect of autism spectrum disorder [17], [18] and prior research has established that autistic traits are normally distributed among the general population [19]. Highly relevant for the current research question, even in non-clinical samples men have more autistic traits than women [16], [20], suggesting that interpersonal interest may be a possible explanation for potential gender differences in quality gift selections. We therefore predict that men have reduced social interest, as measured by the AQ, which can explain potential gender differences in gift selection quality.

The second aspect of interpersonal sensitivity we include in this study is *interpersonal reactivity*, which we measure with the interpersonal reactivity index (IRI) [21]. This scale captures the extent to which one feels empathy and engages in perspective taking. Women tend to score significantly higher than men on this scale, that is, they say that they feel more empathy towards others and are more likely to take the perspective of others [22]. If one is successful in taking the perspective of others, it should be easier to select a good gift for that person, that is why we hypothesize that interpersonal reactivity could also mediate the relationship between gender and gift selection quality.

### Better gift selection through similarity?

Another reason why women think they are better in selecting gifts than men may be that there are simply more gift exchanges between women and women know better what other women want. Caplow [23] found that women are more often the giver but also more often the receiver of a gift. Thus, the majority of gift exchanges are in female-female dyads. As a consequence, women who are asked about their gift giving qualities will be more likely to remember a female-female gift exchange than men are likely to remember a male-male gift exchange. We know from research on preference prediction that predictions for similar others tend to be more accurate than predictions for dissimilar others [24]. It could therefore be the case that women are better at selecting gifts because they give more same-gender gifts than men. We will therefore also investigate whether people are especially good in selecting gifts for someone of the same gender.

### The present studies

The current studies make an important contribution to the literature on gift giving as our studies are the first to use the objective quality of gift giving as a dependent variable. In most other studies on gift giving, gift quality is measured as appreciation by the giver [7], [25], but it is unclear whether self-reports of appreciation reflect true quality or are influenced by social desirability concerns. We therefore introduced a new method in which we asked receivers beforehand which gift (or gift certificate) they would like and assessed whether the selected gift matched the preferred gift.

In our first study, we include actual dyads with different kinds of preexisting relationships (romantic partners, friends, relatives). This enabled us to assess whether the type of relationship between giver and receiver moderates the effect of gender on gift selection quality. In the second and third study we kept the relationship between giver and receiver constant by using strangers as target persons. This setup allowed us to test whether women's gift selection quality is higher in situations in which there is no established relationship. Additionally, in Studies 2 and 3 we test whether interpersonal interest and interpersonal reactivity can explain gender differences in gift selection quality.

## Study 1

### Method

#### Ethics Statement

All three studies reported in this manuscript were conducted at a Dutch University, where institutional review boards or committees are not mandatory, but where researchers need to follow the rules and regulations of the code of conduct for scientific practice, the code of conduct for personal data in scientific research, and the code of ethics for psychologists [26], [27], [28]. Our IRB confirmed the voluntary nature of the ethical approval which applies to our study. The current studies were conducted following the rules and regulations. Specifically, in the first study, the material included a cover page stating the nature of the study and ensuring the anonymity of responses. No data that would identify the participant was recorded. In Studies 2 and 3, participants were psychology students who participate in a number of studies as part of course requirements. The general procedure (including the anonymity of their answers and their right to withdraw from any study) is stated explicitly in the guidelines of the lab, which they can read when they sign up for a certain study. No explicit measures were taken to ensure that participants read these guidelines. When they come to the lab to participate they are informed about the topic and the duration of the specific studies and asked orally whether they want to participate. In our studies, anonymity of the responses was explicitly stated again at the beginning of the study. No personal information of the participants was stored.

#### Participants

Participants were selected from the personal network of the research assistants. In total, 61 dyads participated; 20 dyads with a family relationship (7 male-female dyads, 6 male-male dyads, and 7 female-female dyads), 21 romantic couples (all male-female), and 20 friendship dyads (4 male-male dyads and 16 female-female dyads). Participants mean age was 32.14 years (*SD* = 14.98). Dyad members knew each other on average for 16.92 years (*SD* = 12.98).

#### Procedure & Measures

Participants were visited at home and instructed that this was a study about gift giving. The research assistant made sure that all questionnaires were filled in individually and that the participants could not see the other participant's responses. Participants first answered several demographic questions including their age, gender, their yearly income, and how long they knew their friend/relative/partner. To create a realistic gift selection situation, we designed a booklet with pictures of 30 different typical gifts with a value of around 20 Euros (e.g. a watch, a bottle of champagne, a calendar), which resembled selecting a gift from a catalogue. This set of 30 items was pretested to be equally attractive to males and females. Participants were asked to first select ten gifts from the booklet which they would like to receive for themselves. After that, they were asked to select ten gifts of which they thought their relative/partner/friend would like to receive. We calculated quality of gift selection as the number of gifts chosen by the ‘giver’ that also appeared on the list of the target. This score could vary from 0 to 10.

### Results

#### Do women select better gifts than men?

To analyze whether women select better gift than men, and whether this effect holds for different types of relationships between giver and receiver, we conducted an ANOVA with gender and type of relationship as between subjects factors and the number of correctly selected gifts as dependent variable. (Because the data stem from dyads, the data points from dyad members may not be independent. We therefore ran multilevel models to address this issue but found no significant dyad level variance in the null model only including the random intercept (*p* = .175). Furthermore, a comparison between the model without random intercept (which yielded essentially the same results as the ANOVA) and the model including the random intercept, showed that there was no significant improvement of the −2 log-likelihood score (χ^2^_Change_  = 426.490−425.545 = 0.945). We therefore decided to present the results of the ANOVA because the results are easier to interpret.) The ANOVA revealed a main effect for type of relationship *F*(1, 120) = 4.293, *p* = .016, *η*_p_^2^ = .07, but post hoc tests with Bonferroni correction did not reveal a specific difference between any of the groups, all p's>.10 (*M_Friends_*  =   = 5.10, *SD* = 1.52; *M_Relatives_*  = 5.69, *SD* = 1.56 *M_Romantic partners_*  = 5.79, *SD* = 1.32).

As hypothesized, the analysis also showed a main effect of gender *F*(1, 120) = 4.59, *p* = .034, *η*_p_^2^ = .04 in that women's gift selection (*M* = 5.66, *SD* = 1.38), was better than men's (*M* = 5.33, *SD* = 1.63). There was no significant interaction effect *F*(1, 120) = 1.114, *p* = .332, *η*_p_^2^ = .02, indicating that the effect of gender is independent of the type of relationship between giver and receiver.

#### Better gift selection through similarity?

To investigate the question whether people are especially good in selecting gifts for same gender recipients, we selected the family relationship and friendship dyads because there were no same-sex romantic couples in the sample. An ANOVA with gender of the giver and gender of the receiver as independent variables revealed that there was no interaction effect (*F*(1, 75) = 0.79, *p* = .376), suggesting that people are not better in selecting a gift for a recipient of the same gender than for a recipient of the other gender.

### Discussion

The results of this first study show that women select better gifts than men. This is the first empirical evidence that women are objectively better gift givers. We did not find support for the notion that this effect occurs because women are better in selecting gifts for other women. In the following two studies, we investigate the mediating role of interpersonal sensitivity to explain why women are better gift selectors then men. Study 1 also revealed that the effect of gender is independent of the type of relationship. In the following studies we decided to test our hypothesis under the most minimal condition we could think of: Would women also select better gifts than men when there is no relationship with the receiver?

## Study 2

### Method

#### Participants

Participants were 67 male and 121 female students of Tilburg University who participated in exchange for partial course credit. Their mean age was 21.58 years (*SD* = 3.27).

#### Procedure & Measures

Every participant was seated in a cubicle where the study was conducted via a computer as part of a larger set of unrelated studies. In this study we used a young female as a target person. This target person indicated her preferences prior to the study, so her ratings could be used as an objective standard. Every participant saw a picture of the target person with her name and age next to it. On the same screen, the names of ten different stores were listed. Participants were asked to imagine that they were to buy a gift certificate (worth 10 Euros) of one of the stores for this target person. They were asked to click on the names of the stores in the order of which they thought the target person would want a gift certificate to that store. The ten different stores represented ten different product categories so that every gift certificate would represent a unique kind of gift (fashion (H&M), furniture (IKEA), perfume (Douglas), gardening (Intratuin), books (Selexyz), household (Blokker), hardware (Gamma), toys (Intertoys), music and films (Free Record Shop), liquor (Gall & Gall)).

In order to assess the quality of gift selection we calculated an index for the general correspondence in the rank-orders. This score indicates whether participants have a global picture of the target person's preferences. It was calculated as the absolute difference score between the rank-order of the target and the predicted rank-order for every participant. For example, if the target person likes a gift-certificate to H&M best and a certain participant put H&M on position 3, this would result in a score of 2. Because there are 10 positions the difference score can theoretically vary between 0 (perfect match) and 50 (maximum difference). In this sample, scores ranged between 8 and 44 (*M* = 22.61, *SD* = 5.85).

As described before, we used the AQ to measure interpersonal interest and the IRI to measure interpersonal reactivity. More specifically, we used the abridged version of the AQ, which consists of 28 items [29] (e.g., ‘I would rather go to a library than to a party’). Items were measured with a 7-point-scale and the internal consistency was good (α = .80). The IRI consists of 28 items, measured on a 7-point-scale [21] (e.g., ‘I sometimes try to understand my friends better by imagining how things look from their perspective’) and it had a good internal consistency (α = .79). There was no significant correlation between the IRI and the AQ (*r*(*188*) = −.082, *p* = .261), indicating that these two scales tap into different aspects of interpersonal sensitivity.

### Results

#### Are women better in selecting gifts than men?

To answer this question we investigated whether women are better than men in predicting the target's overall rank-order by comparing their index scores (lower scores  =  better gift giving). An ANOVA with gender as between-subject factor and the score on the gift index as dependent measure revealed that women's rank-orders were closer to the target's rank-order than men's (*M*_women_  = 20.91 (*SD* = 5.33) vs. *M*_men_  = 25.67 (*SD* = 5.52)), *F*(1, 187) = 33.56, *p*<.001, *η*_p_^2^ = .15. This shows that women are better in predicting the target's preferences than men.

#### Why are women better in selecting gifts?

To assess whether differences in interpersonal sensitivity between men and women can explain the differences in quality of gift selection we first established whether the IRI and the AQ had the predicted links with gender and the gift selection index. Consistent with the hypotheses and previous literature, a MANOVA showed that women scored significantly lower on the AQ (*M*_women_  = 2.87, *SD* = 0.53 vs. *M*_men_  = 3.19, *SD* = 0.55); *F*(1, 186) = 14.98, *p*<.001, *η*_p_^2^ = .08) and higher on the IRI (*M*_women_  = 4.71, *SD* = 0.49 vs. *M*_men_  = 4.12, *SD* = 0.47); *F*(1, 186) = 62.08, *p*<.001, *η*_p_^2^ = .25). A regression analysis with the IRI and the AQ as predictors of the gift selection index (*R^2^* = .07, *F*(2, 185) = 6.58, *p* = .002), showed that only the AQ was a significant predictor of the gift selection index (β = .227, *t*(185) = 3.22, *p* = .002), but the IRI was not (β = −.100, *t*(185) = 1.40, *p* = .164). We therefore only included the AQ in our mediation model. We conducted a mediation analysis based on bootstrapping [30] with gender as independent variable, participants' score on the AQ as mediator, and gift selection index as dependent variable. The indirect effect of AQ was significantly different from zero given that the 95% confidence interval did not include 0 (point estimate  = −.08; *BCa* 95% CI between −.19 and −.01). We can therefore conclude that the gender difference in gift selection quality can be better explained by differences in AQ than differences in IRI.

### Discussion

Taken together, the results of Study 2 again support our hypothesis that women are objectively better at selecting gifts than men and that this effect can be partly attributed to differences in social interest. The fact that women show more interest in interpersonal issues seems to explain why they are able to select better gifts for others. Although the results nicely support our hypotheses, there is a certain limitation to this study. This study included only one target person who was a female. Although Study 1 did not support the notion that people are better in predicting the preferences of a similar other, to be sure, and to provide a replication of our findings, we conducted a third study in which we made use of several different target persons, both male and female. The use of several target persons is also a more robust test of our hypotheses because with more data points (in this case more predictions) the reliability of a measure goes up.

## Study 3

### Method

#### Participants

Participants were 28 male and 46 female students at Tilburg University who participated in exchange for partial course credit. Their mean age was 21.33 years (*SD* = 2.42).

#### Procedure & Measures

The procedures were very similar to those of Study 1, but this time participants were asked to rank-order gift certificates for nine different targets (5 male, 4 female) of varying ages (between 20 and 59). These targets had indicated their gift preferences in advance and where unknown to the participants. The participants were presented with a picture, the name, and the age of the target. Interpersonal sensitivity was measured using the IRI (α = .82), measured on a 7-point-scale and the AQ (α = .83), this time was measured with the original 4-point-scale. Again, there was no significant correlation between the IRI and the AQ (*r* = −.185, *p* = .115).

### Results and Discussion

#### Are women better in selecting gifts than men?

To investigate the similarity effect and also test whether there is a main effect of target gender, we calculated the gift selection index separately for male and female targets. We then conducted a repeated measures ANOVA with target gender as a within-subjects factor and participant gender as between-subjects factor. We did not find that women are better in predicting female gift preferences and men are better in predicting male gift preferences as would have been indicated by a significant interaction effect, *F*(1, 72) = 0.67, *p* = .42. We did find two main effects, however, indicating that on average, male targets' gift preferences were predicted more accurately than female targets' gift preferences (*M*_male targets_  = 24.61, *SE* = 0.4232 vs. *M*_female targets_  = 26.11, *SE* = 0.42), *F*(1, 72) = 7.86, *p* = .006, *η*_p_^2^ = .10) and, consistent with our hypothesis, that women are better at predicting gift preferences than men (*M* = 24.71, *SE* = 0.39 vs. *M* = 26.01, *SE* = 0.50), *F*(1, 72) = 4.15, *p* = .045, *η*_p_^2^ = .06. Together, these results show that women are better at predicting others' gift preferences than men for both female and male targets. This indicates that it is not simply projection which drives the effect of Study 2, but that there is a robust difference in the way men and women predict what others would like. In the next step we analyzed whether differences in interpersonal sensitivity can explain this difference.

#### Why are women better in selecting gifts?

Parallel to the analyses in Study 2 we investigated whether the IRI and/or the AQ function as a mediator for the effect reported above. Again, consistent with hypotheses and the previous literature, a MANOVA revealed that women scored significantly lower on the AQ than men (*M* = 1.96, *SD* = 0.32 vs. *M* = 2.17, *SD* = 0.31), *F*(1, 72) = 8.23, *p* = .005, *η*_p_^2^ = .10, and higher on the IRI then men (*M* = 4.64, *SD* = 0.54 vs. *M* = 4.02, *SD* = 0.41), *F*(1, 72) = 26.41, *p*<.001, *η*_p_^2^ = .27. However, again, in the regression analysis with the gift selection index as dependent variable (*R^2^* = .08, *F*(2, 71) = 3.03, *p* = .055), the AQ (β = .285, *t*(71) = 2.46, *p* = .016) was a significant predictor but the IRI was not (β = .044, *t*(71) = 0.38, *p* = .706). We therefore again only included the AQ as a mediator in the model with gender as independent variable and the gift selection index as the dependent variable. Given that we did not find an interaction effect with target gender, we used the overall gift selection index across both target genders for the mediation analysis. In this study the mediating effect of the AQ was only marginally significant, as only the 90% confidence interval did not include 0 (point estimate  = .15, *BCa* 90% CI between .02 and .40).

Because the mediation effect in this study was only marginally significant, we conducted a meta-analysis, combining the datasets from Study 2 and Study 3. This gives us an indication of the robustness of the effects, but should be interpreted with some caution, because the dependent variable is based on one observation per person in Study 2, and nine observations in Study 3. We conducted the mediation analysis as reported below and included the source of the data (Study 2 or Study 3) as a control variable (which did not yield a significant effect in the model). There was a significant effect of gender on gift selection quality (β = .65, t(258) = 5.33, *p*<.001), a significant relationship between gender and the AQ (β = .59, *t*(258) = 4.81, *p*<.001), and a significant relationship between the AQ and gift selection quality (β = .17, *t*(258) = 2.79, *p* = .006). Most importantly, the bootstrap results showed that the indirect effect was significantly larger than 0, because the 95% confidence interval did not include 0 (point estimate  = .10, *BCa* 95% CI between .03 and .21). Based on these results we think that it is safe to conclude that differences in social interest play a role in explaining why women are better at selecting gifts than men.

## General Discussion

The aim of the current studies was to assess whether there are systematic gender differences in gift selection quality. Extending prior research that mostly focused on the consequences of gift giving, after the gift selection has already been made, we focused on the very process of selecting a gift. We assessed who are better gift givers and why. In three studies we show that women select better gifts than men. This finding was observed regardless of the type of relationship between giver and receiver and regardless of the gender of the receiver. Furthermore, the effect was mediated by differences in interpersonal interest and not by differences in interpersonal reactivity.

Our findings add to theory building on gift giving in several ways. First, our research extends earlier work that has argued that gift giving is more important for women. For example, Fischer and Arnold [13] showed that women put more effort in selecting gifts. Our studies suggest that this increased effort may stem from the fact that women have more interest in interpersonal issues in general. More importantly, we showed that this greater involvement actually translates into *better* gift giving. Second, given that men react more negatively to bad gifts from their heterosexual partner than women [12], it is very functional that women are better at selecting gifts. Perhaps, women *need to be better* at giving gifts (at least when buying gifts for their heterosexual partner) in order to avoid the negative consequences of bad gift giving.

### Interpersonal interest versus interpersonal reactivity

We theorized that men and women would differ both in interpersonal interest and interpersonal reactivity and that these differences may explain why women are better at selecting gifts. We indeed found that women score consistently higher on the AQ and the IRI, reflecting differences in interpersonal interest and interpersonal reactivity between men and women. However, only interpersonal interest was a significant mediator between gender and gift selection. One could say that interpersonal interest is a more basic prerequisite for successful social predictions than interpersonal reactivity. If someone is not paying a lot of attention to a social situation, it becomes irrelevant to what extent they possess interpersonal reactivity, as they are less likely to apply it. A possible limitation of our research is that we relied on the IRI and the AQ. Future research may replicate our findings using other measures for interpersonal sensitivity, including experimental manipulations [31], [32], [33], [34], [35].

### Strengths and limitations

We realize that we used dispositional measures to assess interpersonal interest and interpersonal reactivity. The fact that we observed reliable differences on these measures in the predicted direction validates the use of these scales. Yet, there still remains some unexplained variance, so further research may be able to identify additional factors that explain why women select better gifts than men. Given that women report to have more experience with gift giving [13], an additional factor may be that women have learned more about typical preferences. If this is the case, some training may improve men's gift selection. And given that interpersonal interest is more important than interpersonal reactivity, another way to increase the performance of men would be to give them an incentive to do it well [36]. Then again, such incentives may be quite difficult to find, given that our findings were not qualified by the importance of the relationship. Indeed, although we did observe that there was a significant effect for the type of relationship, indicating that for some targets it may be easier to select the right gift, this effect was not more pronounced for men than for women. The samples in our studies are based on university students, so the results are not directly representative to other populations. However, we think that in the general population the gender difference may even be more pronounced, as traditional gender roles are less present at universities and therefore the men in our samples may be less likely to send their girlfriends and wives to buy gifts than men in the general population.

How satisfied people are with a gift may not only depend on their initial preferences but on many other factors like the timing of the gift, the wrapping, and the relationship with the giver. We do not want to argue that these factors are unimportant, but to keep our design simple and objective, we focused on the initial preferences and used the match between these preferences and the choice of the giver as a proxy for gift selection quality. We think that this objectivity has important advantages, as we discuss below.

### Future directions

Our method of using an objective standard of gift quality by asking receivers beforehand which gifts they like takes away social desirability concerns in gift giving research and is therefore a straightforward measure of predictive accuracy. There are numerous other aspects of the giver, the situation, and the relationship between giver and receiver that may influence gift selection quality. Although not the main focus of the current investigation, our first study shows for example that there was a main effect of type of relationship. This effect needs to be investigated further because the analysis did not have enough power to reveal the specific differences between groups. Our design can also be easily extended to investigate other factors that may influence gift selection quality. For example, it may be interesting to see how time pressure or the presence of another person influences gift selection quality and whether these factors affect men and women in the same way.

Another interesting venue for future research may be the integration of our findings with recent findings on false beliefs in gift giving. Two recent sets of studies show that givers tend to have different beliefs about the quality of certain types of gifts than receivers do, possibly leading to suboptimal gift selection. For example, givers believe that more expensive gifts are appreciated more than less expensive gifts, whereas receivers do not share this view [7]. Receivers also prefer gifts they explicitly ask for over gifts they did not ask for, whereas givers think that unrequested gifts will be appreciated as much as requested gifts [25]. Finally, when givers have to select gifts for several different independent recipients they tend to select different gifts for every recipient, even if every recipient likes the same gift best [37]. These studies show that givers base their gift selection on several false beliefs. The question is whether everyone is equally susceptible to these beliefs or whether they maybe have a stronger influence on women because they contemplate more over the gift they choose. If this is the case, false beliefs may moderate the effect of gender on gift selection quality, such that the gender effect is attenuated when false beliefs come into play. The method we present in this paper lends itself to investigate these questions. Only if gift selection quality is investigated using an objective measure can we be sure that the receiver does not base his or her evaluation of the gift on prior expectations or beliefs.

## Conclusions

The exchange of gifts is of great importance in almost every culture. Many studies have investigated what motivates gift giving [9], [38]. We took a different approach and instead focused on the quality of the gift: we investigated if people are able to select the right gift. Our results show that, when it comes to selecting the best gift for others, women do a better job than men and this gender difference is due to the interpersonal interest women have in others. Or, to quote a lay-person's answer to the question of who gives better gifts: “I would say women do because women actually think about the gift they are giving. Guys don't.” [4].
